# Investigations into the Intimate Structure of the Pancreas

**Published:** 1869-09-15

**Authors:** 


### FOREIGN ITEMS.

### *Investigations into the Intimate Structure of the Pancreas.*

BY DR. GIANNUZZI.

It is generally believed that the structure of the pancreas is identical with that of the salivary glands, but my investigations have demonstrated the contrary, and it may be readily perceived that there are marked differences.

I hope to present to the Academy (of Sciences, Paris), at an early day, my memoir, *in extenso*, with appropriate illustrations. From this time I may say:

1st. The excretory ducts of the pancreas have very thin walls, which are lined interiorly with cylindrical epithelium. They have not the same connection with the secretory vesicles as in the salivary glands, but they establish around them a network composed of very fine tubes, which have no epithelium, and which surround with their meshes the pancreatic cells. This network may be compared to that of the bile ducts of the liver.

2d. The network of the excretory ducts of the different vescicles, which form the same glandular lobule, have inter-connections and constitute a common system.

3d. The blood vessels of the pancreas follow, generally, by means of their terminal ramifications, the course of the pancreatic duct. They surround the vescicles and the glandular lobules by their capillaries, which are interposed within the meshes of the pancreatic ducts.

4th. The pancreatic vescicles have no walls.

5th. The pavement epithelium of the vescicles is formed of flattened cells with nuclei and prolongations. They are, in fact, very similar to those of the salivary glands. However, the nuclei are readily seen, and the proto-plasma is more granular and contains fatty granulations.

6th. I have not found, in the glandular vescicles of the dog, upon which I made my investigations, the semi-lunar bodies which I discovered for the first time, in the sub-maxillary gland of the same animal (Berichte d. Koen. Sachs. Geselsch. der Wiss. Sitz., Nov. 25, 1865). The presence of these bodies has been confirmed and again found in other animals by MM. Kölliker, Heidenheim and Boll.

The injections of the pancreatic ducts were made with Prussian Blue, and those of the blood-vessels with gelatine and carmine. The apparatus which I used was the continuous-pressure apparatus of M. Ludwig. I was obliged, always, to use very little force; at most that which would be produced by a column of nine or ten centimetres of mercury.

